# MicroRNAs in Human Placental Development and Pregnancy Complications

**DOI:** 10.3390/ijms14035519

**Published:** 2013-03-08

**Authors:** Guodong Fu, Jelena Brkić, Heyam Hayder, Chun Peng

**Affiliations:** Department of Biology, York University, Toronto, Ontario M3J 1P3, Canada; E-Mails: guodong@yorku.ca (G.F.); brkicj@yorku.ca (J.B.); heyam23@yorku.ca (H.H.)

**Keywords:** microRNA, human placenta, proliferation, migration, invasion, angiogenesis, preeclampsia

## Abstract

MicroRNAs (miRNAs) are small non-coding RNAs, which function as critical posttranscriptional regulators of gene expression by promoting mRNA degradation and translational inhibition. Placenta expresses many ubiquitous as well as specific miRNAs. These miRNAs regulate trophoblast cell differentiation, proliferation, apoptosis, invasion/migration, and angiogenesis, suggesting that miRNAs play important roles during placental development. Aberrant miRNAs expression has been linked to pregnancy complications, such as preeclampsia. Recent research of placental miRNAs focuses on identifying placental miRNA species, examining differential expression of miRNAs between placentas from normal and compromised pregnancies, and uncovering the function of miRNAs in the placenta. More studies are required to further understand the functional significance of miRNAs in placental development and to explore the possibility of using miRNAs as biomarkers and therapeutic targets for pregnancy-related disorders. In this paper, we reviewed the current knowledge about the expression and function of miRNAs in placental development, and propose future directions for miRNA studies.

## 1. Introduction

MicroRNAs are small endogenous single-stranded RNAs that post-transcriptionally regulate the expression of various target genes. In 1993, the first miRNA, lin-4, was discovered as a 22-nucleotide non-coding RNA that regulated the timing of post-embryonic development by repressing lin-14 protein expression in *Caenorhabditis elegans*[1]. The second miRNA discovered, let-7, was also identified in nematodes [2]. Both miRNAs function in a similar way by binding with partial complementarity to the 3′-untranslated region (UTR) of their target genes. To date, more than a 1000 human miRNAs have been identified, each potentially controlling hundreds of target genes. It is now well established that these tiny miRNAs act as important gene regulators to control various physiological events, including placental development.

The placenta is a transient organ during pregnancy. It serves as the interface between the fetal and maternal environments and is involved in the exchange of gases, nutrients and waste products between the mother and the growing fetus [3]. Moreover, the placenta functions as an endocrine organ producing a number of pregnancy-associated hormones and growth factors to regulate fetal growth and maternal physiology [4,5]. Finally, placenta acts as a barrier to protect the fetus from maternal immune attack [6]. Proper placental development is vital for embryo survival and health [7]. Defects in the function and development of the placenta have been associated with many pregnancy complications, such as preeclampsia (PE) [8,9], intrauterine growth restriction (IUGR) [10,11], small-for-gestational-age (SGA) [12,13], preterm birth (PTB) [12,14], and gestational diabetes mellitus (GDM) [15].

Increasing evidence suggests that miRNAs are important regulators of placental development [12,14,16–20]. Many miRNAs have been identified in human placental tissues [21]. In addition, core proteins required for miRNA biogenesis have also been detected in villous trophoblast cells [22]. *In vitro* studies have shown that miRNAs regulate trophoblast cell proliferation, migration, invasion, apoptosis, and angiogenesis [16,19,20,23,24]. Aberrant expression of miRNAs in placenta from women with compromised pregnancies has been reported [14,16,25,26]. Therefore, it is likely that miRNAs are necessary for the normal development of the placenta and abnormal expression of miRNAs is associated with defective placentation and compromised pregnancies [17,18]. In this article, we reviewed the current state of miRNA research in human placenta, focusing primarily on miRNA expression, regulation, and functions, as well as its potential involvement in pregnancy-associated disorders, particularly PE.

## 2. Key Processes in Human Placental Development

**Figure 1:**
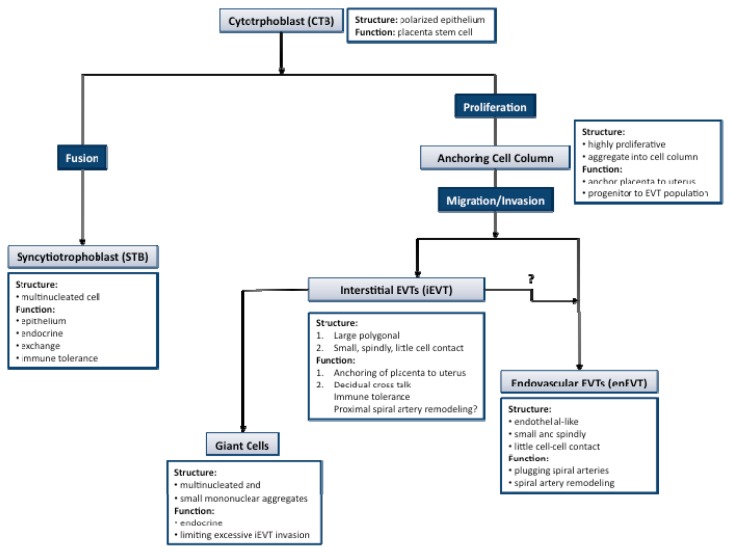
Trophoblast differentiation. The cytotrophoblast progenitor (CTBs) cells line the basement membrane of the placental villi where they differentiate into two general pathways: villous and extravillous trophoblasts. In the villous pathway, CTBs fuse into multinucleated syncytiotorphoblasts (STBs). From the proliferative anchoring columns, trophoblasts differentiate into two subpopulations of extravillous trophoblasts—interstitial (iEVTs) and endovascular (enEVTs). The iEVTs invade the decidua and the inner third of the myometrium while enEVTs are intimately involved in remodeling the maternal spiral arteries. There is evidence iEVT may differentiate into enEVTs in the superficial part of the decidua.

Human placental development begins with the implantation of the blastocyst [27]. The initial adhesion of the blastocyst to the decidua, followed by establishment of more stable attachments and invasion into the implantation site is all centered around the interactions between cytotrphoblastic cells of the trophectoderm and the decidualized uterus [28,29]. Cells of the trophectoderm undergo temporally and spatially regulated differentiation as they continue to invade the entire decidualized endometrium up until the inner third of the myometrium along with the maternal vasculature [27]. The cytotrophoblast progenitor cells line the basement membrane of the placental villi where they differentiate into two general pathways: villous and extravillous trophoblasts (Figure 1) [30,31]. *In vitro* trophoblast cultures have shown that both differentiation pathways occur spontaneously.

In the villous pathway, the mononucleated cytotrophoblasts (CTBs) fuse into multinucleated syncytiotrophoblasts (STBs) forming the syncytial layer that covers the placental villous tree. These cells are intimately involved in the exchange of gases, nutrients and waste across the materno-fetal interface [32]. The syncytial layer also plays a major role in the maintenance of pregnancy through the production of pregnancy-related hormones, such as human gonadotropin (hCG) and human placental lactogen (hPL) [33]. Additionally, STBs are in direct contact with the maternal blood and therefore are required to exhibit a level of immune tolerance [34]. The syncytium is non-proliferative and therefore is continually replenished throughout pregnancy through the fusion of the underlying progenitor cell layer [35,36].

In the extravillous pathway, CTBs from the cell column of the anchoring villi, exit the cell cycle, and shift from a proliferative phase into a migratory and invasive phenotype [37]. These invasive cells are termed extravillous cytotrphoblasts (EVTs) and can be further subdivided into interstitial EVTs (iEVT) and endovascular EVTs (enEVT) that appear to have distinct roles in the maternal decidua. The iEVTs have two distinct phenotypes: large polygonal iEVTs that secure the placenta to the uterus and small spindle-shaped iEVTs that invade deep into the decidua [38]. The invasive iEVTs show a distinct expression profile of adhesion molecules and human leukocyte antigen (HLA) class I major histocompatibility complex antigens [39–41]. The iEVTs secrete not only proteases that facilitate the breakdown of the decidual extracellular matrix, but also protease inhibitors, suggesting a self-regulating role in their invasive capacity [42]. Furthermore, as iEVTs invade the deeper portions of the decidua they form into placental bed giant cells capable of producing hormones and protease inhibitors, suggesting a role in pregnancy maintenance and in limiting EVT invasion past the myometrium, respectively [43].

A key event during placental development is the remodeling of the spiral arteries from high-resistance, low flow muscular vessels to sac-like vessels of low-resistance and high-flow [44–46]. This process involves cross-talk between different cell types with enEVTs as the key players. The enEVTs invade the maternal vessels and travel down the lumen in a retrograde manner [44,45], replacing the endothelial cells of the maternal vessels through a process known as pseudovasculogenesis or vascular mimicry [39,47,48]. Endovascular EVTs have the ability to exist within the maternal vasculature like an endothelial cell mainly due to their switch from an epithelial to endothelial adhesion molecule profile [30]. The remodeling of maternal spiral arteries continues until mid second trimester, and goes as far as the inner third of the myometrium [44]. The resulting remodeled spiral arteries have an increased length and lumen diameter and are unresponsive to vaso-constrictive agents [30,49]. This new, looser phenotype is an essential part of healthy pregnancies since it provides the necessary flow of maternal blood into the intervillous spaces to sustain the growing demands of the fetus throughout gestation [30].

The placenta is a highly vascularized organ composed of both maternal and embryonic blood vessels. From the fetal side, two arteries and one vein from the umbilical cord feed into the placenta supplying vessels to each cotyledon where they further branch in the chorionic villi forming capillary loops [48]. On the maternal side, the EVTs are involved in remodeling the maternal spiral arteries, which leads to the increased blood flow toward the fetomaternal interface. The vascular density of the cotyledons, as well as uterine and umbilical blood flows greatly increase in the later part of gestation coinciding with an exponential increase in fetal growth [50]. Adequate placental vascularization is essential in order to keep pace with the growing fetus.

In addition to differentiation, migration, invasion and angiogenesis, proliferation and apoptosis also play a role in placental development. Proliferation is mostly confined to the CTB progenitor cell layer and is a vital process in the formation of anchoring columns in early gestation that attach the placenta to the uterine wall. Interestingly, proliferative markers decline later in gestation coinciding with a reduction in placental growth [51]. On the other hand, proliferative markers in the STB layer are not detected at any stage of pregnancy. However, apoptosis is important in STB turnover [52]

All key processes of placental development are temporally and spatially regulated throughout pregnancy. Disruption of these events results in improper development and function of the placenta and contributes to gestational complications. For example, preeclamptic placentas show a decrease in invasive EVTs, insufficient remodeling of the spiral arteries as well as an increase in apoptotic cells [27,53]. On the other hand, studies of IUGR placenta demonstrate a decrease in villi vascular density and capillaries in the stroma, reduced number of terminal villi and overall smaller placental size and volume due to reduced proliferation [54,55]. Similarly, placentas from SGA pregnancies have reduced villous development [56]. The potential role of miRNAs in these processes and their dysregulation in pregnancy complications are the focus of this review.

## 3. Expression of MiRNAs in Human Placenta

It is now well documented that miRNAs can be produced by human placenta. Using microarray, Northern blot hybridization, *in situ* hybridization and real-time PCR techniques, many miRNAs have been detected in trophoblast cells and placental tissues [12,14,57]. Key molecules involved in miRNA biogenesis, such as Drosha, Exportin 5, Dicer, Argonaute 2 (Ago2) and DP103, have also been identified in trophoblast cells [22,58], confirming that the miRNA biogenesis pathway is active in human placenta. Moreover, miRNA expression in the placenta is regulated by environment factors [22], signaling pathways [59], and epigenetic modification [60].

### 3.1. MicroRNA Biogenesis and Mechanism of Gene Regulation

MicroRNA biogenesis comprises miRNA gene transcription and several post-transcriptional modifications that result in the maturation of miRNAs. Firstly, miRNA genes are transcribed into primary transcripts (pri-miRNAs) by RNA polymerase II (pol II) [61,62]. The pri-miRNAs are capped with 7-methylguanosine and are polyadenylated [62]. Secondly, the pri-miRNAs are cleaved into precursor miRNAs (pre-miRNAs) by the nuclear RNase III enzyme Drosha and its coactivator DGCR8 [63]. The pre-miRNAs are about 70 nt in length and have a characteristic imperfect stem-loop structure [63]. Thirdly, the pre-miRNAs are transported from the nucleus into the cytoplasm by Exportin 5 and are subject to cleavage by a second RNase III enzyme Dicer to produce a miRNA duplex [64]. Finally, the RNA duplex is unwound and the miRNA serving as a guide molecule is then loaded into the miRNA-induced silencing complex (RISC) for target recognition [65–68].

The mature miRNAs control gene expression at the post-transcriptional level, by repressing protein translation of target genes and/or inducing target mRNA degradation. Regulation by miRNA is largely dependent on complementary binding of its “seed sequence” (nucleotides 2–8) to target sites in the 3′UTR of mRNAs. Imperfect binding of miRNA to the partially complementary sequences in the 3′UTRs of the target mRNAs leads to repression of protein translation [61,69–72] whereas near perfect binding of miRNAs to the complementary sites of 3′UTR leads to cleavage and degradation of target mRNAs [73,74]. In addition to 3′ UTR, some miRNAs can also bind to target sites in the 5′ UTR [75]. Finally, it has also been suggested that miRNAs suppress gene expression in proliferating cells but enhance gene expression in quiescent cells [76].

### 3.2. Detection and Identification of MiRNAs in Placenta

Recognition of miRNAs as a novel class of gene regulators began with the cloning and sequencing of more than 100 novel endogenous miRNAs of 21–25 bp long from nematodes, flies and mammals [77–79]. To date, cloning of endogenously expressed short RNAs is still a powerful tool for miRNA discovery. Deep sequencing is a high-throughput technology for miRNA identification and expression profiling [80]. Due to its high-sensitivity, deep sequencing can also be used to reveal multiple miRNA variants with heterogeneous ends, lengths and expression levels [81].

Many other detection methods have been applied to miRNA studies, such as Northern blot analyses [22,57], quantitative real-time PCR [19,22,82], microarrays [21,26,57,83] and *in situ* hybridization (ISH) [12,84]. Northern blot assays are used, not only to detect the levels of miRNAs, but also to provide insightful information on the distribution of individual miRNA precursors in different tissues. A limitation of this method is low sensitivity and specificity because miRNAs have very short sequences. Utilization of locked nucleic acid (LNA) probes where every third nucleotide of the probe is substituted with LNAs greatly improves the sensitivity and specificity of Northern blotting for miRNA detection [85,86]. RT-PCR is a widely used method for assessing levels of miRNA expression. Real time PCR allows for analyzing levels of miRNAs in a large number of clinical samples, such as placental biopsies, plasma or serum, due to its high sensitivity.

MicroRNA microarray is a powerful high-throughput assay capable of profiling the expression of large number of miRNAs at the same time [80,87]. This technique has been extensively used to compare miRNA expression levels between normal placenta and placenta from compromised pregnancies [18,26,83]. Results from microarray experiments usually require validation and confirmation using larger sample sizes and more sensitive techniques, such as real-time PCR.

MicroRNA ISH is a useful method to investigate the localization of miRNA expression in cells or tissues as well as the relative abundance of miRNAs [88,89]. However, the sensitivity of this detection is low because binding affinity to target miRNA is weak with the normal DNA or RNA probes. The LNA modified ISH probes are able to increase the affinity as well as the sensitivity and specificity of this assay [88,89]. Using an LNA probe specific for miR-210, ISH revealed that miR-210 is primarily expressed in both villous trophoblasts and iEVTs [12]. ISH has also been used to detect the expression of miR-517b in STBs and villous stroma cells [82].

The above mentioned miRNA detection methodologies have their own advantages and limitations. The intrinsic properties of miRNAs, such as small size and large number of closely related family members with highly similar sequences, make it challenging to measure miRNAs accurately. Ideally, more than one method should be used in one study to increase the reliability of the results.

### 3.3. MicroRNA Expression during Different Stages of Placental Development

Recent studies have shown that a large number of miRNAs are expressed in human placenta and some miRNA genes are expressed in a temporal and/or tissue-specific manner during different stages of placental development in line with their functions in regulating placental development and trophoblast cell activities.

Many miRNAs are expressed in human placenta and some of them, such as the C19MC and C14MC clusters, are specifically or preferentially expressed in the placenta [90,91]. The C19MC, located in chromosome 19q13.41, is the largest miRNA cluster identified to date [80,91–93]. This cluster harbors 46 pre-miRNAs transcribed from a non-protein-coding host gene and expressed only in the placenta [93]. The C19MC is primate specific and expressed from the paternal allele [94]. On the other hand, the C14MC cluster, containing 46 miRNAs in 14q32, is also highly expressed in the human placenta but is encoded by maternally imprinted genes [60]. The imprinted genes are usually activated at critical developmental stages and involved in controlling cell differentiation and fate in the embryonic growth or placenta tissues [95].

Expression levels of some miRNAs vary with the stages of placental development. Using TaqMan Array a recent study shows that the level of C19MC miRNAs in trophoblast cells increases significantly from first to third trimester while C14MC miRNA levels have the opposite pattern [90]. A small increase in miRNA levels within miR-371-3 cluster, which is adjacent to C19MC, from the first to third trimester has also been observed [90]. In addition to these miRNA clusters, the levels of many other miRNAs in primary cultures of trophoblast cells also vary significantly between first trimester and term placenta [90]. In our recent studies, we found that miR-378a-5p levels were higher in first and second trimester than in third trimester placenta [19] while miR-376c levels were higher in second and third trimesters than that in the first trimester [16]. Our finding that placental miR-376c levels are higher in the third trimester than in the first trimester is conflicting with the qPCR array results reported by Morales-Prieto *et al*. [90]. The discrepancy may be due to the use of different materials to extract RNA. Placental tissue samples were used in our study whereas the other study used primary cultures of purified trophoblast cells.

Differential expression of miRNAs during different gestational stages suggests that miRNAs are regulated developmentally and that they have stage-specific functions during pregnancy. However, more studies are needed to fully characterize the spatial and temporal expression pattern of each individual miRNA in the placenta.

### 3.4. Regulation of MiRNA Expression in Placenta

How miRNA expression is regulated in human placenta is not well understood. Oxygen tension, which plays critical roles in placental development [96–98], has been shown to be a major regulator of miRNA expression in placenta. Microarray analysis reveals that many miRNAs in primary cultures of trophoblast cells are regulated by hypoxia. Further analysis using real-time PCR confirms that miR-93, -205, -224, -335, -451, and -491 are upregulated while miR-424 is down-regulated when cells were cultured under 1% oxygen for 48 h [57].

MicroRNA-210 is well known as a sensor of hypoxia [99,100]. The miR-210 gene is located within the intron of the hypoxia-inducible *AK123483* gene [101]. The level of miR-210 increases in response to low oxygen tension in many different cell types and it is up-regulated in hypoxia associated diseases, such as cancer and PE [99]. In trophoblast cells, miR-210 levels are also strongly upregulated by hypoxia [12].

Several studies suggest that the changes of miRNA levels in response to hypoxia are due to transcriptional regulation of the miRNA genes. Expression of Ago2 and its interacting protein, DP-103, was not affected by hypoxia, suggesting that hypoxia regulation of miRNA expression unlikely occurs at the level of miRNA processing [22]. On the other hand, many hypoxia-responsive transcription factors that bind to hypoxia-responsive elements (HREs) on target gene promoters [102] have been identified to regulate miRNA gene expression in placenta. A major hypoxia-responsive transcription factor is the hypoxia inducible factor-1 alpha (HIF-1α) [99]. HIF-1α is directly recruited to the HRE region in miR-210 promoter, which leads to induction of miR-210 expression [103]. In turn, miR-210 stabilizes HIF-1α by targeting glycerol-3-phosphate dehydrogenase 1-like (GPD1L) mRNA [104]. GPD1L is an enzyme that causes HIF-1α hyperhydroxylation thus decreasing HIF-1α stability [104]. In addition to HIF-1α, p50, a subunit of the hypoxia-responsive transcription factor nuclear factor kappa-B (NFκB), also mediates the effect of hypoxia on miR-210 expression in trophoblast cells [105].

Members of the activating protein-1 (AP-1) transcription factor family are expressed in human placenta, particularly in EVTs [106]. These transcription factors are implicated in trophoblast cell proliferation and differentiation, as well as pregnancy complications, such as PE [107]. Recently, an AP-1 site along with NFκB binding sites, has been identified in the miR-155 promoter [108]. Treatment of HTR8/SVneo cells with lipopolysaccharides (LPS) resulted in an increase in miR-155 levels. LPS activated JunB and FosB, two AP-1 members, as well as NFκB. Luciferase reporter assays further revealed that AP-1 and NFκB stimulated the transcription of miR-155 [108].

Evidence is emerging to suggest that signaling molecules and environmental toxins are also involved in the control of miRNA expression in trophoblast cells. Leukemia inhibitory factor (LIF), which is known to be a major regulator of trophoblast functions, has been reported to upregulate miR-21 and miR-93, but down-regulate miR-141, expression in JEG-3 cells [109]. In addition, endocrine disruptors, such as Bisphenol A (BPA), significantly increased the expression of several miRNAs in trophoblast cell lines, HTR8 and A3. The increase in miR-146a levels after treatment with BPA was further validated by real-time PCR [110]. Similarly, miR-146a is down-regulated in TCL-1 cells exposed to nicotine and benzo(a)pyrene [111]. In addition, miR-16, miR-21 and miR-146a levels are also significantly lower in placentas obtained from women who smoked during pregnancy than in placentas from non-smoking women [111]. These findings provide new insights into how environmental toxins may affect placental and fetal development.

Epigenetic regulation, particularly DNA methylation, has been shown to play a key role in controlling tissue-specific expression of placental miRNAs. The C19MC miRNAs are not detectable in most cells but treatment with a demethylation agent strongly increased their expression in cancer cells [112]. In addition, the expression pattern of these miRNAs is highly correlated with the methylation status of a CpG-rich region, located upstream of the C19MC cluster. This CpG-rich region is hypomethylated in placenta but hypermethylated in other cells, suggesting that methylation plays a key role in the placenta-specific expression of C19MC and that demethylation is involved in the activation of C19MC in cancer cells [112]. Similarly, the expression of the C14MC cluster miRNAs in placenta is also regulated by a distal intergenic, Germ-Line-Derived differentially methylated region (DMR) [60].

## 4. Regulation of Placental Development and Function by MiRNAs

Recent studies have revealed that miRNAs exert regulatory effects on trophoblast cell proliferation, apoptosis, migration and invasion, as well as on angiogenesis. Some miRNAs also regulate trophoblast cell metabolism (Table 1). These findings suggest that miRNAs can regulate placental development and function. However, only a small number of miRNAs have been studied so far and the functions for the majority of placental miRNAs remain unknown.

### 4.1. MicroRNAs and Trophoblast Cell Proliferation and Apoptosis

MicroRNAs have been shown to regulate trophoblast cell proliferation and apoptosis during placental development (Table 1). In the first trimester placental explants, siRNA-mediated knockdown of Dicer significantly enhanced cytotrophoblast proliferation as well as the expression of two pro-mitogenic signaling molecules within cytotrophoblasts, ERK and SHP-2 [58], suggesting that dicer-dependent miRNAs mainly exert negative effects on cell proliferation during early pregnancy, in part through inhibition of growth factors signaling. However, studies on individual miRNAs demonstrate that some miRNAs promote while others suppress trophoblast cell proliferation and survival.

Several miRNAs have been reported to enhance trophoblast cell proliferation and/or survival. For example, miR-378a-5p increased proliferation and survival of HTR-8/SVneo cells and enhanced the outgrowth of first trimester placental explants [19]. One of the target genes confirmed to mediate the effect of miR-378a-5p in trophoblast cells is Nodal, a transforming growth factor (TGF-β) family member known to inhibit proliferation and to promote apoptosis in trophoblast cells [119–121]. However, miR-378a-5p has a stronger effect than siRNA targeting Nodal in enhancing cell proliferation and survival [19], indicating that additional genes are involved in miR-378a-5p-regulated trophoblast cell activities. Validated target genes of miR-378a-5p characterized in other cell types include aromatase [122], Fus-1 and SuFu [123]. Whether or not these genes are involved in miR-378a-5p-regulated trophoblast cell activities remains to be investigated. Similar to miR-378a-5p, miR-376c also promotes trophoblast cell proliferation and survival partially by regulating Nodal and TGF-β signaling as it represses the expression of activin receptor-like kinase 5 (ALK5) and ALK7, type I receptors for TGF-β and Nodal, respectively [16]. MicroRNA-141 was highly expressed in first trimester trophoblast cells [92]. Silencing of miR-141 completely inhibited the proliferation of JEG-3 cells, suggesting that miR-141 promotes trophoblast cell proliferation [109].

On the other hand, several miRNAs have been shown to inhibit trophoblast cell proliferation. Among them, miRNA-155 inhibits cell proliferation by suppressing cyclin D1 [24]. MicroRNA-675, encoded by the imprinted H19 gene, also exerts inhibitory effects on trophoblast cell proliferation. In human choriocarcinoma cell line, JEG-3, inhibition of miR-675 or silencing of H19 promoted cell proliferation [115]. Since miR-675 inhibited Nodal Modulator-1 (NOMO-1) expression and overexpression of NOMO-1 rescued the antiproliferative effect of miR-675 [115], it is possible that a mechanism by which miR-675 inhibits trophoblast cell proliferation is to promote Nodal signaling by down-regulating NOMO-1. In addition, targeted deletion of miR-675 in mice resulted in a significant increased in placenta size, accompanied by an increase in miR-675 target genes, including insulin-like growth factor-1 (IGF-1) receptor [124]. IGF-1 is known to promote proliferation and inhibit apoptosis in human trophoblast cells [125], therefore, miR-675 could inhibit placental growth by suppressing IGF signaling. Moreover, miR-29b has been shown to induce apoptosis of trophoblast cells partly due to the down-regulation of the Bcl-2 family member MCL1 expression [23]. On the other hand, miR-182 was shown to have an anti-apoptotic effect on trophoblast cells [83], potentially by targeting FoxO3a and subsequently down-regulating Bim levels [126].

### 4.2. MicroRNAs and Trophoblast Cell Migration and Invasion

As discussed above, migration and invasion of EVTs to the decidua and myometrium are critical events during placentation [127]. Dicer is abundantly expressed in EVTs of first trimester placenta [58], suggesting miRNAs play important roles in trophoblast invasion. Indeed, recent studies have documented a number of miRNAs that are involved in the regulation of trophoblast cell migration and invasion (Table 1).

Some miRNAs, such as miR-195 [20], miR-376c [16], and miR-378a-5p [19], have been reported to enhance trophoblast migration/invasion. Interestingly, these miRNAs all target Nodal/TGF-β signaling, which has been shown to inhibit trophoblast invasion [119,128,129]. For example, miR-195 increased trophoblast cell invasion in part via modulating the expression of ActRIIA [20], a type II receptor for several members of the TGF-β superfamily, including Nodal [130]. MicroRNA-376c induced trophoblast invasion and migration and explants outgrowth by targeting ALK7 and ALK5 to impair Nodal/TGF-β signaling [16]. On the other hand, miR-378a-5p promoted trophoblast migration and invasion by directly inhibiting the expression of Nodal [19].

Several miRNAs, including miR-210 [105], miR-34a [84,131] and miR-29b [23,84] have inhibitory effects on trophoblast cell invasion. It was reported that miR-210 inhibited the migration and invasion capability of trophoblast cells by repressing Ephrin-A3 and Homeobox-A9 [105]. The inhibiting effect of miR-34a on trophoblast cell invasion was mediated through inhibition of Notch1 and Jagged1 signaling [131] and down-regulation of plasminogen activator inhibitor-1 (PAI-1) [84]. On the other hand, miR-29b reduced trophoblast cell invasion probably via targeting matrix metalloproteinase (MMP)-2 and integrin β1 (ITGB1) in trophoblast cell lines [23].

Thus, miRNAs can exert positive and negative effects on trophoblast cell migration and invasion by modulating the activity of signaling pathways, enzymes, and adhesion molecules. Again, only a small fraction of the miRNAs expressed in the placenta has been investigated for their functions. For each individual miRNA, only one or a few target genes have been characterized. More studies are needed to fully understand the function and mechanism of miRNAs in controlling these processes.

### 4.3. MicroRNAs and Placental Angiogenesis

Direct evidence supporting the role of miRNAs in placental vascularization and spiral artery remodeling is limited. However, several studies have implicated miRNAs in regulating these processes. Dicer is abundantly expressed in the first trimester EVTs and in the perivascular villous stroma, suggesting a potential role for miRNAs in spiral artery remodeling [58]. In addition, Dicer has also been found in the villous and umbilical vascular endothelial and smooth muscle cells [132]. The important role of miRNAs in angiogenesis is further supported by the findings that Dicer deletion resulted in embryonic lethality, with compromised blood vessel formation [133].

MicroRNA-20b targets ephrin receptor B4 (EPHB4) and ephrin-B2 [134], which are known to play important roles in placental angiogenesis [135]. However, miR-20b was not detected in endothelial cells of term placenta by ISH [136], and the direct effect of miR-20b on angiogenesis has not yet been determined. MicroRNA-29b represses trophoblast cell invasion and tube-formation, as well as the expression of vascular endothelial growth factor-A (VEGFA) [23]. As VEGFA is known to be a key regulator of placental angiogenesis [137], these findings suggest that miR-29b has an inhibitory effect on angiogenesis. Similarly, miR-16 also targets VEGFA and the expression level of VEGFA is negatively correlated with the level of miR-16 expression in patients with severe PE [118]. In addition, overexpression of miR-16 in decidua-derived mesenchymal stem cells inhibits the ability of HTR8 cells to migrate and human umbilical vein endothelial cells (HUVEC) to form tube-like structures [118], suggesting that miR-16 is a negative regulator of angiogenesis.

Although not yet studied for their roles in placental angiogenesis, many miRNAs have been demonstrated to regulate angiogenesis in tumor cells. For example, miR-125b has been reported to target VE-Cadherin and reduce endothelial cell tube formation as well as placental growth factor (PlGF) levels [138,139]. On the other hand, miR-21 promotes cancer cell angiogenesis by up-regulating HIF-1α and VEGF expression through the activation of AKT and ERK 1/2 [140]. Similarly, miR-93 promotes tumor angiogenesis in U87 cells by targeting integrin-β8 [141]. Interestingly, integrin-β8 deficiency was shown to be lethal in mouse embryos in part due to insufficient vascularization of the placenta and yolk sac [142]. Moreover, miR-378a-5p enhances tumor angiogenesis [123]. Since these miRNAs and their targets are also expressed in human placenta, it is possible that they may also regulate angiogenesis during placental development. However, this remains to be directly tested using placental and uterine tissues. Placental and uterine tissue co-culture system has been established [143,144] and it will be a useful model to study miRNA functions in placental angiogenesis and vessel remodeling.

## 5. Implication of MiRNAs in Compromised Pregnancies

Aberrant expression of miRNAs has been detected in various pregnancy complications, such as PE, PTB, SGA, IUGR, and GDM (Table 2). Some recent studies have shed lights into the mechanisms by which miRNAs contribute to the pathogenesis of PE.

### 5.1. Aberrant Expression of MiRNAs in Preeclampsia

Preeclampsia is a unique multisystem disease of human pregnancy characterized by maternal hypertension and proteinuria [147]. Severe PE is a major cause of maternal morbidity and accompanies adverse perinatal outcomes, such as perinatal death, PTB, SGA, and IUGR [8,148]. A key pathological feature of PE is composed of shallow invasion of EVTs into the maternal decidua and myometrium, poor transformation of maternal spiral arteries and subsequent placental underperfusion [45,46,149]. Recent studies suggest that miRNAs are involved in the physiological regulation and pathological development of PE.

Many studies have been done to compare miRNA expression profiles between preeclamptic and normal placentas [14,18,25,26,132,136]. A number of miRNAs have been found to be up- or down-regulated in preeclamtic placentas. The placental miRNAs that have been identified as expressed significantly different between healthy and preeclamptic patients are summarized in Table 2. It should be noted that discrepancies exist with respect to which miRNAs are aberrantly expressed in PE placenta. For example, miR-20a and miR-17 were found to be up-regulated in preeclampsia by qPCR but not by miRNA microarray analysis [136]. Similarly, qPCR detected a significant decreased in miR-378a-5p levels in preterm placentas [19] and in miR-376c [16] and miR-675 [115] levels in term placentae, as compared to their gestational age-matched controls. However, microarray-based miRNA profiling studies did not detect significant changes in these miRNAs in PE placentas. On the other hand, the levels of some miRNAs were found to be significantly altered in PE placentas by microarray, but not by real-time PCR. Furthermore, while many studies have reported miR-210 as one of the most strongly upregulated miRNAs in PE (Table 2), one report showed that miR-210 expression was not significantly changed in placentas from PE patients as compared to normotensive patients by real-time PCR [14]. Finally, some miRNAs identified to be down- or up-regulated by different studies are not consistent. MicroRNA-195 was found to be significantly up-regulated in placenta from women with severe PE by microarray analysis followed by qRT-PCR [25]. However, other studies showed a decrease of miR-195 [20,26] in preeclamptic pregnancies. These discrepancies may be due to the different sensitivity of detection methods, different gestational stages of samples used, and/or individual variations. Furthermore, it may be attributed to the sampling sites within the placenta, as only a tiny portion of placenta is often used in RNA sample preparation.

### 5.2. Potential Contribution of MiRNAs to Preeclampsia

Some of the dysregulated miRNAs in PE have been examined for their functional roles in placenta and findings suggest that they contribute to the pathogenesis of PE by altering key processes in placental development. Specifically, aberrant expression of miRNAs could lead to limited proliferation and shallow invasion of trophoblast cells, as well as insufficient remodeling of maternal spiral arteries and defective angiogenesis. For example, several up-regulated miRNAs in preeclamptic placenta, including miR-210 [83,105], miR-20b [136], miR-29b [23], miR-16 [25], miR-155 [117] and miR-675 [115], have been demonstrated or suggested to inhibit angiogenesis and/or trophoblast proliferation, migration and invasion. On the other hand, a number of down-regulated miRNAs, such as miR-378a-5p [19], miR-376c [16], and miR-195 [20], promote trophoblast cell proliferation, survival, and/or invasion.

Using primary trophoblast cells prepared from placenta with severe PE, it was found that elevated expression of miR-210 is responsible for mitochondrial dysfunctionin part due to its ability to suppress the expression of iron-sulfur cluster scaffold homologue (ISCU) (Table 1) [12,113]. MicroRNA-210 and another up-regulated miRNA in preeclamptic placenta, miR-518c, have been reported to target hydroxysteroid (17-beta) dehydrogenase 1 (HSD17B1), a steroidogenetic enzyme expressed in the placenta and responsible for the conversion of estrone to 17β-estradiol [114]. Interestingly, a prospective cohort study reveals that plasma HSD17B1 are significantly lower in pregnant women at 20 to 23 and 27 to 30 weeks of gestation before the onset of PE, as compared with healthy pregnant women, suggesting that plasma HSD17B1 can be used as a potential prognostic factor for PE [114].

### 5.3. MicroRNAs and Other Gestational Disorders

IUGR is characterized as failure of a fetus to reach its growth potential and it can be caused by maternal, fetal, placental, and external factors [10,11]. Other gestational disorders include PTB [12,14], SGA [12,13,83,116], and GDM [15,145]. Emerging evidence suggests that miRNAs are dysregulated in these disorders (Table 2). Interestingly, a recent study demonstrates that expression levels of seven placenta-specific miRNAs (miR-518b, miR-1323, miR-516b, miR-515-5p, miR-520h, miR-519d, and miR-526b) were significantly reduced in placentas from IUGR pregnancies than in placentas from uncomplicated pregnancies [146]. Low levels of miR-21 and miR-16 in the placenta are associated with fetal growth and reduced levels of these miRNAs have both shown to be predictive of SGA status [116]. Aberrant expression of miRNAs in these gestational disorders suggested a role for miRNAsin pregnancy complications. Further investigation of their target genes may reveal the molecular pathogenesis of these diseases as well as identify potential therapeutic targets and diagnostic biomarkers.

### 5.4. Circulating MiRNA as Potential Biomarkers for Pregnancy and Pregnancy-Associated Disorders

MicroRNAs produced by human trophoblast cells can be secreted into maternal plasma or serum through an exosome-mediated pathway [82] and have the potential to serve as biomarkers [150,151]. Some placental miRNAs (miR-516-5p, -517*, -518b, -520a*, -520h, -525 and -526a) have been detected in maternal plasma and recognized as pregnancy associated miRNAs [152]. Both miR-21 and miR-141 are highly expressed in placenta tissue and they are abundantly detected in maternal circulation [82,153]. Some miRNA levels are much higher in plasma from pregnant women than that from non-pregnant women [152,154]. In addition, some miRNAs (miR-141, -149, -299-5p, and -135b) showed higher concentrations in the maternal plasma before delivery than after delivery [153]. Furthermore, some miRNA (*i.e.*, miR-141) levels in plasma increase with the progression of gestation [153]. Hence, the levels of some placental miRNAs in maternal plasma are specifically associated with pregnancy and gestational age and may offer a noninvasive means for monitoring the status or condition of pregnancy.

Altered circulation levels of miRNAs have been reported in compromised pregnancies. Our recent studies show that plasma miR-376c levels are lower in PE patients than in control subjects. Interestingly, the decrease in plasma miR-376c levels are found at 16–18 weeks of pregnancy, in a group of subjects who later developed PE, suggesting the possibility of using miR-376c as a predictive biomarker for PE [16]. The functional significance of placental miRNA releasing into maternal circulation is not well understood. It is possible that these circulating miRNAs are involved in mediating maternal-fetal communication and maternal adaptation of pregnancy. This possibility remains to be tested.

Gestational Diabetes Mellitus (GDM) is one type of diabetes that occurs during pregnancy and significantly increases the risk of a number of adverse consequences for the fetus and mother. Serum miRNAs were analyzed by collecting blood samples of pregnant women at their 16–19 gestational weeks, who were diagnosed as GDM at 25–28 gestational weeks [145]. Three miRNAs (miR-132, -29a and -222) were significantly decreased in GDM women with respect to the controls in similar gestational weeks [145], shedding light on miRNAs as the possible targets of earlier intervention and diagnoses.

Taken together, miRNAs in maternal circulation may serve as a new class of biomarkers for noninvasive prenatal diagnosis, gestational stage monitoring or early detection of pregnancy-associated diseases. However, more studies are needed, particularly using a larger number of samples from different gestational stages, to confirm if circulating miRNAs can be used as biomarkers for pregnancy.

## 6. Summary and Perspectives

The human placenta produces miRNAs and secretes them into maternal circulations [82,150,151,153]. Several lines of evidence support the notion that miRNAs are critical players in human placental development. First, miRNA expression in the placenta varies with gestational stages and is regulated by environmental factors, such as oxygen tension [11,105]. Second, functional analysis revealed that miRNAs regulate various events associated with placental development and function, including trophoblast cell proliferation, migration, invasion, and angiogenesis, as well as cellular metabolism and steroid hormone production [12,16,19,23,114]. Third, gestational disorders, particularly PE, are associated with aberrant expression of miRNAs [14,18,26,136].

Despite these exciting progresses on miRNA research in human placenta, our understanding on how miRNAs contribute to normal placental development and function and placental pathologies is very limited. Future studies should continue to explore the regulation of miRNA expression in placenta. Many transcription factors are known to control trophoblast cell differentiation and placental development [155]. Do they regulate miRNA expression in the placenta? Similarly, various signaling pathways are known to be critical in regulating placental physiology but their roles in regulating miRNA expression are poorly understood. MicroRNAs potentially target many genes and a gene is often targeted by multiple miRNAs. Therefore, it will be important to identify a network of genes targeted by a miRNA and how these target genes mediate the effect of the miRNA in placenta. It will be also interesting to investigate how different miRNAs interact to regulate the expression of a target gene. These studies will enhance our understanding of the molecular mechanisms underlying miRNA regulation of trophoblast activity. Current functional studies depend heavily on the use of trophoblast cell lines and primary cultures of trophoblast cells. To fully understand the functions of miRNAs, *in vivo* models are needed. The use of tissue biopsies from normal and compromised pregnancies will also provide valuable information on miRNA functions and their involvement in the pathogenesis of pregnancy-related disorders.

Detection of miRNAs in the maternal circulation raises the possibility of using miRNAs as biomarkers to monitor the progression of normal pregnancy and gestational diseases. Aberrant expression of miRNAs in placenta from compromised pregnancies also suggests the potential of using miRNAs as therapeutic targets. Future studies should continue to identify novel candidate miRNAs that may be of etiological relevance to gestational disorders. Specifically, it will be important to collect blood samples from a large number of pregnant women who have healthy or complicated pregnancies and screen for miRNA candidates to predict gestational disorders. To explore the possibility of using miRNAs to prevent or treat gestational disorders, strategies to increase or decrease miRNA levels in the placenta will need to be developed and *in vivo* studies using animal models should be carried out to determine if over- or under-expression of a particular miRNA might prevent the development of gestational disorders.

## Figures and Tables

**Table 1:** The function and targets of MicroRNAs (miRNAs) in trophoblast.

miRNA	Function	Target genes	Cells tested	References
miR-210	↓ migration/invasion	*EFNA3*, *HOXA9*	CTBs (1st trimester)	[105]
	↓ iron metabolism	*ISCU*	BeWo, Swan71, placental tissue	[12]
	↓ mitochondrial respiration	*ISCU*	Primary trophoblasts	[113]
	↓ steroid metabolism	*HSD17B1*	BeWo	[114]

miR-376c	↑ proliferation/invasion/migration	*ALK5*, *ALK7*	HTR8/SVneo, placental explants	[16]

miR-378a-5p	↑ proliferation/invasion/migration	*NODAL*	HTR8/SVneo, placental explants	[19]

miR-195	↑ invasion	*ActRIIA*	HTR8/SVneo	[20]

miR-675	↓ proliferation	*NOMO-1*	JEG-3	[115]

miR-21	↑ proliferation/invasion	*PTEN*	TCL-1	[90,116]

miR-155	↓ proliferation/migration	*CCND1*	HTR8/SVneo	[117]

miR-16	↓ proliferation	*CCNE1*	dMSC	[118]
	↓ invasion		HTR8/SVneo	
	
	↓ angiogenesis		HUVEC	

miR-34a	↓ proliferation/invasion	*NOTCH1, JAG1*	JAR	[119]

miR-29b	↑ apoptosis	*MCL1*, *MMP2, VEGFA*, *ITGB1*	HTR8/SVneo, BeWo	[23]
	↓ invasion			
	↓ angiogenesis			

*EFNA3*, Ephrin-A3; *HOXA9*, Homeobox protein Hox-A9; *ISCU*, Iron-sulfur cluster scaffold; *HSD17B1*, Hydroxysteroid (17-beta) dehydrogenase 1; *ALK5*, Activin receptor-like kinase 5; *ALK7*, Activin receptor-like kinase 7; *ActRIIA*, Activin Receptor IIA; *NOMO-1*, NODAL modulator 1; *PTEN*, Phosphatase and tensin homolog; *CCND1*, Cyclin D1; *CCNE1*, Cyclin E1; *JAG1*, JAGGED 1; *MCL1*, Myeloid cell leukemia sequence 1; *MMP2*, Matrix metalloproteinase-2; *VEGFA*, Vascular endothelial growth factor A; *ITGB1*, integrin beta 1; ↓, Inhibit; ↑, Induce.

**Table 2:** Aberrant expression of miRNAs in compromised pregnancy.

Compromised pregnancy	Sample	Regulation	miRNAs	Method of detection	References
PE	Placenta	Up-regulated	miR-20b	Microarray & qRT-PCR	[136]
			miR-16, miR-29b, miR-195, miR-26b, miR-181a, miR-335 and miR-222		[25]
			miR-210, miR-152 and miR-518b		[26]

			miR-516a-5p, miR-512-3p, miR-2277 and miR-524-3p	Microarray	[136]

			miR-182 and miR-210	qRT-PCR	[12,83]
			miR-17 and miR-20a		[136]
			miR-155		[117]

			miR-210, miR-193b, miR-144*, miR-193*, miR-18a, miR-185, miR-19a, miR-590-5p, miR-142-3p, miR-451, miR-22*, miR-526b*, miR-520a-3p, miR-10b, miR-20a, miR-518f*, miR-146b-5p, miR-517c, miR-518c, miR-5258-5p, miR-519e* and miR-126*	High-throughput sequencing & qRT-PCR-based array	[114]

	Plasma	Up-regulated	miR-210	qRT-PCR	[105]

	Placenta	Down-regulated	miR-18a, miR-411, miR-377, miR-363 and miR-542-3p	Microarray & qRT-PCR	[26]

			miR-101, miR-10b, miR-218, miR-590, miR-204, miR-32, miR-126*, miR-19a, miR-154*, miR-625, miR-144, miR-195, miR-150, miR-1, miR-18b and miR-450 miR-151-3p, miR-146a, miR-192 and miR-34c-5p	Microarray	[26]
					[136]

			miR-376c	qRT-PCR	[16]
			miR-378a-5p		[19]
			miR-195		[20]
			miR-675		[115]

	Plasma	Down-regulated	miR-376c	qRT-PCR	[16]
SGA	Placenta	Up-regulated	miR-210	qRT-PCR	[12]

		Down-regulated	miR-16 and miR-21	qRT-PCR	[116]

PE + SGA	Placenta	Up-regulated	miR-210	qRT-PCR	[12,83]

PTB	Fetal membranes	Up-regulated	miR-25, miR-338, miR-101, miR-449, miR-154, miR-135a, miR-142-3p, miR-202* and miR-136	Microarray	[132]

			miR-338, miR-449, miR-136 and miR-199a*	qRT-PCR	[132]

GDM	Serum	Down-regulated	miR-132, miR-29a and miR-222	qRT-PCR	[145]

IUGR	Placenta	Down-regulated	miR-518b, miR-1323, miR-516b, miR-515-5p, miR-520h, miR-519d and miR-526b	qRT-PCR	[146]

PE, Preeclampsia; SGA, Small-for-gestational-age; PTB, Preterm Birth; GDM, Gestational Diabetes Mellitus; IUGR, Intrauterine Growth Restriction. Placenta-specific miRNAs are underlined.

## References

[1] Lee R.C., Feinbaum R.L., Ambros V. (1993). The *C. elegans* heterochronic gene lin-4 encodes small RNAs with antisense complementarity to lin-14. Cell.

[2] Reinhart B.J., Slack F.J., Basson M., Pasquinelli A.E., Bettinger J.C., Rougvie A.E., Horvitz H.R., Ruvkun G. (2000). The 21-nucleotide let-7 RNA regulates developmental timing in *Caenorhabditis elegans*. Nature.

[3] Cross J.C. (1998). Formation of the placenta and extraembryonic membranes. Annu. N. Y. Acad. Sci.

[4] Murphy V.E., Smith R., Giles W.B., Clifton V.L. (2006). Endocrine regulation of human fetal growth: The role of the mother, placenta, and fetus. Endocr. Rev.

[5] Dey S.K., Lim H., Das S.K., Reese J., Paria B.C., Daikoku T., Wang H. (2004). Molecular cues to implantation. Endocr. Rev.

[6] Pijnenborg R. (2002). Implantation and immunology: Maternal inflammatory and immune cellular responses to implantation and trophoblast invasion. Reprod. Biomed. Online.

[7] Rossant J., Cross J.C. (2001). Placental development: Lessons from mouse mutants. Nat. Rev. Genet.

[8] Steegers E.A., von Dadelszen P., Duvekot J.J., Pijnenborg R. (2010). Pre-eclampsia. Lancet.

[9] James J.L., Whitley G.S., Cartwright J.E. (2010). Pre-eclampsia: Fitting together the placental, immune and cardiovascular pieces. J. Pathol.

[10] Zhong Y., Tuuli M., Odibo A.O. (2010). First-trimester assessment of placenta function and the prediction of preeclampsia and intrauterine growth restriction. Prenat. Diagn.

[11] Mouillet J.F., Chu T., Hubel C.A., Nelson D.M., Parks W.T., Sadovsky Y. (2010). The levels of hypoxia-regulated microRNAs in plasma of pregnant women with fetal growth restriction. Placenta.

[12] Lee D.C., Romero R., Kim J.S., Tarca A.L., Montenegro D., Pineles B.L., Kim E., Lee J., Kim S.Y., Draghici S. (2011). MiR-210 targets iron-sulfur cluster scaffold homologue in human trophoblast cell lines: Siderosis of interstitial trophoblasts as a novel pathology of preterm preeclampsia and small-for-gestational-age pregnancies. Am. J. Pathol.

[13] Saenger P., Czernichow P., Hughes I., Reiter E.O. (2007). Small for gestational age: Short stature and beyond. Endocr. Rev.

[14] Mayor-Lynn K., Toloubeydokhti T., Cruz A.C., Chegini N. (2011). Expression profile of microRNAs and mRNAs in human placentas from pregnancies complicated by preeclampsia and preterm labor. Reprod. Sci.

[15] Gauster M., Desoye G., Tötsch M., Hiden U. (2012). The placenta and gestational diabetes mellitus. Curr. Diab. Rep.

[16] Fu G., Ye G., Nadeem L., Ji L., Manchanda T., Wang Y., Zhao Y., Qiao J., Wnag Y.-L., Lye S. (2013). MicroRNA-376c impairs transforming growth factor-beta and nodal signaling to promote trophoblast cell proliferation and invasion. Hypertension.

[17] Mouillet J.F., Chu T., Sadovsky Y. (2011). Expression patterns of placental microRNAs. Birth Defects Res. A.

[18] Enquobahrie D.A., Abetew D.F., Sorensen T.K., Willoughby D., Chidambaram K., Williams M.A. (2011). Placental microRNA expression in pregnancies complicated by preeclampsia. Am. J. Obstet. Gynecol..

[19] Luo L., Ye G., Nadeem L., Fu G., Yang B.B., Honarparvar E., Dunk C., Lye S., Peng C. (2012). MicroRNA-378a-5p promotes trophoblast cell survival, migration and invasion by targeting Nodal. J. Cell Sci.

[20] Bai Y., Yang W., Yang H., Liao Q., Ye G., Fu G., Ji L., Xu P., Wang H., Li Y. (2012). Downregulated miR-195 detected in preeclamptic placenta affects trophoblast cell invasion via modulating ActRIIA expression. PLoS One.

[21] Barad O., Meiri E., Avniel A., Aharonov R., Barzilai A., Bentwich I., Einav U., Gilad S., Hurban P., Karov Y. (2004). MicroRNA expression detected by oligonucleotide microarrays: System establishment and expression profiling in human tissues. Genome Res.

[22] Donker R.B., Mouillet J.F., Nelson D.M., Sadovsky Y. (2007). The expression of Argonaute2 and related microRNA biogenesis proteins in normal and hypoxic trophoblasts. Mol. Hum. Reprod.

[23] Li P., Guo W., Du L., Zhao J., Wang Y., Liu L., Hu Y., Hou Y. (2013). MicroRNA-29b contributes to pre-eclampsia through its effects on apoptosis, invasion and angiogenesis of trophoblast cells. Clin. Sci.

[24] Dai Y., Qiu Z., Diao Z., Shen L., Xue P., Sun H., Hu Y. (2012). MicroRNA-155 inhibits proliferation and migration of human extravillous trophoblast derived HTR-8/SVneo cells via down-regulating cyclin D1. Placenta.

[25] Hu Y., Li P., Hao S., Liu L., Zhao J., Hou Y. (2009). Differential expression of microRNAs in the placentae of Chinese patients with severe pre-eclampsia. Clin. Chem. Lab. Med.

[26] Zhu X.M., Han T., Sargent I.L., Yin G.W., Yao Y.Q. (2009). Differential expression profile of microRNAs in human placentas from preeclamptic pregnancies vs normal pregnancies. Am. J. Obstet. Gynecol..

[27] Norwitz E.R. (2007). Defective implantation and placentation: Laying the blueprint for pregnancy complications. Reprod. Biomed. Online.

[28] Red-Horse K., Zhou Y., Genbacev O., Prakobphol A., Foulk R., McMaster M., Fisher S.J. (2004). Trophoblast differentiation during embryo implantation and formation of the maternal-fetal interface. J. Clin. Invest.

[29] Clancy K.B. (2009). Reproductive ecology and the endometrium: Physiology, variation, and new directions. Am. J. Phys. Anthropol.

[30] Anin S., Vince G., Quenby S. (2004). Trophoblast invasion. Hum. Fert.

[31] Knofler M. (2010). Critical growth factors and signalling pathways controlling human trophoblast invasion. Int. J. Dev. Biol.

[32] Mayhew T.M. (2012). Estimating oxygen diffusive conductances of gas-exchange systems: A stereological approach illustrated with the human placenta. Ann. Anat..

[33] Guibourdenche J., Fournier T., Malassiné A., Evain-Brion D. (2009). Development and hormonal functions of the human placenta. Folia Histochem. Cytobiol.

[34] Nakamura O (2009). Children’s immunology, what can we learn from animal studies (1): Decidual cells induce specific immune system of feto-maternal interface. J. Toxicol. Sci.

[35] Richart R. (1961). Studies of placental morphogenesis. I. Radioautographic studies of human placenta utilizing tritiated thymidine. Proc. Soc. Exp. Biol. Med.

[36] Kar M., Ghosh D., Sengupta J. (2007). Histochemical and morphological examination of proliferation and apoptosis in human first trimester villous trophoblast. Hum. Reprod.

[37] Kemp B., Kertschanska S., Kadyrov M., Rath W., Kaufmann P., Huppertz B. (2002). Invasive depth of extravillous trophoblast correlates with cellular phenotype: A comparison of intra- and extrauterine implantation sites. Histochem. Cell Biol.

[38] Huppertz B. (2007). The feto-maternal interface: Setting the stage for potential immune interactions. Semin. Immunopathol.

[39] Damsky C.H., Fisher S.J. (1998). Trophoblast pseudo-vasculogenesis: Faking it with endothelial adhesion receptors. Curr. Opin. Cell Biol.

[40] Damsky C.H., Fitzgerald M.L., Fisher S.J. (1992). Distribution patterns of extracellular matrix components and adhesion receptors are intricately modulated during first trimester cytotrophoblast differentiation along the invasive pathway, *in vivo*. J. Clin. Invest.

[41] Trowsdale J., Moffett A. (2008). NK receptor interactions with MHC class I molecules in pregnancy. Semin. Immunol.

[42] Fisher S.J., Leitch M.S., Kantor M.S., Basbaum C.B., Kramer R.H. (1985). Degradation of extracellular matrix by the trophoblastic cells of first-trimester human placentas. J. Cell. Biochem.

[43] Al-Lamki R.S., Skepper J.N., Burton G.J. (1999). Are human placental bed giant cells merely aggregates of small mononuclear trophoblast cells? An ultrastructural and immunocytochemical study. Hum. Reprod.

[44] Cartwright J.E., Fraser R., Leslie K., Wallace A.E., James J.L. (2010). Remodelling at the maternal-fetal interface: Relevance to human pregnancy disorders. Reproduction.

[45] Kaufmann P., Black S., Huppertz B. (2003). Endovascular trophoblast invasion: Implications for the pathogenesis of intrauterine growth retardation and preeclampsia. Biol. Reprod.

[46] Pijnenborg R., Vercruysse L., Hanssens M. (2006). The uterine spiral arteries in human pregnancy: Facts and controversies. Placenta.

[47] Zhou Y., Fisher S.J., Janatpour M., Genbacev O., Dejana E., Wheelock M., Damsky C.H. (1997). Human cytotrophoblasts adopt a vascular phenotype as they differentiate. A strategy for successful endovascular invasion?. J. Clin. Invest.

[48] Khankin E.V., Royle C., Karumanchi S.A. (2010). Placental vasculature in health and disease. Semin. Thromb. Hemost.

[49] Kam E.P.Y., Gardner L., Loke Y.W., King A. (1999). The role of trophoblast in the physiological change in decidual spiral arteries. Hum. Reprod.

[50] Reynolds L.P., Redmer D.A. (2001). Angiogenesis in the Placenta. Biol. Reprod.

[51] Danihel L., Gomolcák P., Korbel M., Pruzinec J., Vojtassák J., Janík P., Babál P. (2002). Expression of proliferation and apoptotic markers in human placenta during pregnancy. Acta Histochem.

[52] Huppertz B. (2010). IFPA award in placentology lecture: Biology of the placental syncytiotrophoblast—Myths and facts. Placenta.

[53] Allaire A.D., Ballenger K.A., Wells S.R., McMahon M.J., Lessey B.A. (2000). Placental apoptosis in preeclampsia. Obstet. Gynecol.

[54] Chen C.P., Bajoria R., Aplin J.D. (2002). Decreased vascularization and cell proliferation in placentas of intrauterine growth-restricted fetuses with abnormal umbilical artery flow velocity waveforms. Am. J. Obstet. Gynecol.

[55] Krebs C., Macara L.M., Leiser R., Bowman A.W., Greer I.A., Kingdom J.C. (1996). Intrauterine growth restriction with absent end-diastolic flow velocity in the umbilical artery is associated with maldevelopment of the placental terminal villous tree. Am. J. Obstet. Gynecol.

[56] Jackson M.R., Walsh A.J., Morrow R.J., Mullen J.B., Lye S.J., Ritchie J.W. (1995). Reduced placental villous tree elaboration in small-for-gestational-age pregnancies: Relationship with umbilical artery Doppler waveforms. Am. J. Obstet. Gynecol.

[57] Mouillet J.F., Chu T., Nelson D.M., Mishima T., Sadovsky Y. (2010). MiR-205 silences MED1 in hypoxic primary human trophoblasts. FASEB J.

[58] Forbes K., Farrokhnia F., Aplin J.D., Westwood M. (2012). Dicer-dependent miRNAs provide an endogenous restraint on cytotrophoblast proliferation. Placenta.

[59] Ji L., Brkić J., Liu M., Fu G., Peng C., Wang Y.L. (2012). Placental trophoblast cell differentiation: Physiological regulation and pathological relevance to preeclampsia. Mol. Aspects Med..

[60] Seitz H., Royo H., Bortolin M.L., Lin S.P., Ferguson-Smith A.C., Cavaillé J. (2004). A large imprinted microRNA gene cluster at the mouse Dlk1-Gtl2 domain. Genome Res.

[61] Bartel D.P. (2004). MicroRNAs: Genomics, biogenesis, mechanism, and function. Cell.

[62] Cai X., Hagedorn C.H., Cullen B.R. (2004). Human microRNAs are processed from capped, polyadenylated transcripts that can also function as mRNAs. RNA.

[63] Lee Y., Ahn C., Han J., Choi H., Kim J., Yim J., Lee J., Provost P., Rådmark O., Kim S. (2003). The nuclear RNase III Drosha initiates microRNA processing. Nature.

[64] Lund E., Güttinger S., Calado A., Dahlberg J.E., Kutay U. (2004). Nuclear export of microRNA precursors. Science.

[65] Denli A.M., Tops B.B., Plasterk R.H., Ketting R.F., Hannon G.J. (2004). Processing of primary microRNAs by the Microprocessor complex. Nature.

[66] Gregory R.I., Yan K.P., Amuthan G., Chendrimada T., Doratotaj B., Cooch N., Shiekhattar R. (2004). The microprocessor complex mediates the genesis of microRNAs. Nature.

[67] Guo H., Ingolia N.T., Weissman J.S., Bartel D.P. (2010). Mammalian microRNAs predominantly act to decrease target mRNA levels. Nature.

[68] He L., Hannon G.J. (2004). MicroRNAs: Small RNAs with a big role in gene regulation. Nat. Rev. Genet.

[69] Bartel D.P. (2009). MicroRNAs: Target recognition and regulatory functions. Cell.

[70] Huang Y., Zou Q., Song H., Song F., Wang L., Zhang G., Shen X. (2010). A study of miRNAs targets prediction and experimental validation. Protein Cell.

[71] Nilsen T.W. (2007). Mechanisms of microRNA-mediated gene regulation in animal cells. Trends Genet.

[72] Zhao S., Liu M.F. (2009). Mechanisms of microRNA-mediated gene regulation. Sci. China C Life Dci.

[73] Valencia-Sanchez M.A., Liu J., Hannon G., Parker R. (2006). Control of translation and mRNA degradation by miRNAs and siRNAs. Genes Dev.

[74] Zeng Y., Yi R., Cullen B.R. (2003). MicroRNAs and small interfering RNAs can inhibit mRNA expression by similar mechanisms. Proc. Natl. Acad. Sci. USA.

[75] Lytle J.R., Yario T.A., Steitz J.A. (2007). Target mRNAs are repressed as efficiently by microRNA-binding sites in the 5′ UTR as in the 3′ UTR. Proc. Natl. Acad. Sci. USA.

[76] Vasudevan S., Tong Y., Steitz J.A. (2007). Switching from repression to activation: MicroRNAs can up-regulate translation. Science.

[77] Lee R.C., Ambros V. (2001). An extensive class of small RNAs in *Caenorhabditis elegans*. Science.

[78] Lau N.C., Lim L.P., Weinstein E.G., Bartel D.P. (2001). An abundant class of tiny RNAs with probable regulatory roles in *Caenorhabditis elegans*. Science.

[79] Lagos-Quintana M., Rauhut R., Lendeckel W., Tuschl T. (2001). Identification of novel genes coding for small expressed RNAs. Science.

[80] Landgraf P., Rusu M., Sheridan R., Sewer A., Iovino N., Aravin A., Pfeffer S., Rice A., Kamphorst A.O., Landthaler M. (2007). A mammalian microRNA expression atlas based on small RNA library sequencing. Cell.

[81] Guo L., Yang Q., Lu J., Li H., Ge Q., Gu W., Bai Y., Lu Z. (2011). A comprehensive survey of miRNA repertoire and 3′ addition events in the placentas of patients with pre-eclampsia from high-throughput sequencing. PLoS One.

[82] Luo S.S., Ishibashi O., Ishikawa G., Ishikawa T., Katayama A., Mishima T., Takizawa T., Shigihara T., Goto T., Izumi A. (2009). Human villous trophoblasts express and secrete placenta-specific microRNAs into maternal circulation via exosomes. Biol. Reprod..

[83] Pineles B.L., Romero R., Montenegro D., Tarca A.L., Han Y.M., Kim Y.M., Draghici S., Espinoza J., Kusanovic J.P., Mittal P. (2007). Distinct subsets of microRNAs are expressed differentially in the human placentas of patients with preeclampsia. Am. J. Obstet. Gynecol.

[84] Umemura K., Ishioka S., Endo T., Ezaka Y., Takahashi M., Saito T. (2013). Roles of microRNA-34a in the pathogenesis of placenta accreta. J. Obstet. Gynaecol. Res.

[85] Tolstrup N., Nielsen P.S., Kolberg J.G., Frankel A.M., Vissing H., Kauppinen S. (2003). OligoDesign: Optimal design of LNA (locked nucleic acid) oligonucleotide capture probes for gene expression profiling. Nucleic Acids Res.

[86] Varallyay E., Burgyan J., Havelda Z. (2008). MicroRNA detection by northern blotting using locked nucleic acid probes. Nat. Protoc.

[87] Miska E.A., Alvarez-Saavedra E., Townsend M., Yoshii A., Sestan N., Rakic P., Constantine-Paton M., Horvitz H.R. (2004). Microarray analysis of microRNA expression in the developing mammalian brain. Genome Biol.

[88] Kloosterman W.P., Wienholds E., de Bruijn E., Kauppinen S., Plasterk R.H. (2006). *In situ* detection of miRNAs in animal embryos using LNA-modified oligonucleotide probes. Nat. Methods.

[89] Wienholds E., Kloosterman W.P., Miska E., Alvarez-Saavedra E., Berezikov E., de Bruijn E., Horvitz H.R., Kauppinen S., Plasterk R.H. (2005). MicroRNA expression in zebrafish embryonic development. Science.

[90] Morales-Prieto D.M., Chaiwangyen W., Ospina-Prieto S., Schneider U., Herrmann J., Gruhn B., Markert U.R. (2012). MicroRNA expression profiles of trophoblastic cells. Placenta.

[91] Donker R.B., Mouillet J.F., Chu T., Hubel C.A., Stolz D.B., Morelli A.E., Sadovsky Y. (2012). The expression profile of C19MC microRNAs in primary human trophoblast cells and exosomes. Mol. Hum. Reprod.

[92] Bentwich I., Avniel A., Karov Y., Aharonov R., Gilad S., Barad O., Barzilai A., Einat P., Einav U., Meiri E. (2005). Identification of hundreds of conserved and nonconserved human microRNAs. Nat. Genet.

[93] Bortolin-Cavaillé M.L., Dance M., Weber M., Cavaillé J. (2009). C19MC microRNAs are processed from introns of large Pol-II, non-protein-coding transcripts. Nucleic Acids Res.

[94] Noguer-Dance M., Abu-Amero S., Al-Khtib M., Lefèvre A., Coullin P., Moore G.E., Cavaillé J. (2010). The primate-specific microRNA gene cluster (C19MC) is imprinted in the placenta. Hum. Mol. Genet.

[95] Lewis A., Mitsuya K., Umlauf D., Smith P., Dean W., Walter J., Higgins M., Feil R., Reik W. (2004). Imprinting on distal chromosome 7 in the placenta involves repressive histone methylation independent of DNA methylation. Nat. Genet.

[96] Genbacev O., Joslin R., Damsky C.H., Polliotti B.M., Fisher S.J. (1996). Hypoxia alters early gestation human cytotrophoblast differentiation/invasion *in vitro* and models the placental defects that occur in preeclampsia. J. Clin. Invest.

[97] Caniggia I., Winter J., Lye S.J., Post M. (2000). Oxygen and placental development during the first trimester: Implications for the pathophysiology of pre-eclampsia. Placenta.

[98] Adelman D.M., Gertsenstein M., Nagy A., Simon M.C., Maltepe E. (2000). Placental cell fates are regulated *in vivo* by HIF-mediated hypoxia responses. Genes Dev.

[99] Chan S.Y., Loscalzo J. (2010). MicroRNA-210: A unique and pleiotropic hypoxamir. Cell Cycle.

[100] Devlin C., Greco S., Martelli F., Ivan M. (2011). MiR-210: More than a silent player in hypoxia. IUBMB Life.

[101] Camps C., Buffa F.M., Colella S., Moore J., Sotiriou C., Sheldon H., Harris A.L., Gleadle J.M., Ragoussis J. (2008). hsa-miR-210 Is induced by hypoxia and is an independent prognostic factor in breast cancer. Clin. Cancer Res.

[102] Cummins E.P., Taylor C.T. (2005). Hypoxia-responsive transcription factors. Pflugers Arch.

[103] Kulshreshtha R., Ferracin M., Wojcik S.E., Garzon R., Alder H., Agosto-Perez F.J., Davuluri R., Liu C.G., Croce C.M., Negrini M. (2007). A microRNA signature of hypoxia. Mol. Cell. Biol.

[104] Kelly T.J., Souza A.L., Clish C.B., Puigserver P. (2011). A hypoxia-induced positive feedback loop promotes hypoxia-inducible factor 1alpha stability through miR-210 suppression of glycerol-3-phosphate dehydrogenase 1-like. Mol. Cell. Biol.

[105] Zhang Y., Fei M., Xue G., Zhou Q., Jia Y., Li L., Xin H., Sun S. (2012). Elevated levels of hypoxia-inducible microRNA-210 in pre-eclampsia: New insights into molecular mechanisms for the disease. J. Cell. Mol. Med.

[106] Bamberger A.M., Bamberger C.M., Aupers S., Milde-Langosch K., Löning T., Makrigiannakis A. (2004). Expression pattern of the activating protein-1 family of transcription factors in the human placenta. Mol. Hum. Reprod.

[107] Marzioni D., Todros T., Cardaropoli S., Rolfo A., Lorenzi T., Ciarmela P., Romagnoli R., Paulesu L., Castellucci M. (2010). Activating protein-1 family of transcription factors in the human placenta complicated by preeclampsia with and without fetal growth restriction. Placenta.

[108] Dai Y., Diao Z., Sun H., Li R., Qiu Z., Hu Y. (2011). MicroRNA-155 is involved in the remodelling of human-trophoblast-derived HTR-8/SVneo cells induced by lipopolysaccharides. Hum. Reprod.

[109] Morales-Prieto D.M., Schleussner E., Markert U.R. (2011). Reduction in miR-141 is induced by leukemia inhibitory factor and inhibits proliferation in choriocarcinoma cell line JEG-3. Am. J. Reprod. Immunol.

[110] Avissar-Whiting M., Veiga K.R., Uhl K.M., Maccani M.A., Gagne L.A., Moen E.L., Marsit C.J. (2010). Bisphenol A exposure leads to specific microRNA alterations in placental cells. Reprod. Toxicol.

[111] Maccani M.A., Avissar-Whiting M., Banister C.E., McGonnigal B., Padbury J.F., Marsit C.J. (2010). Maternal cigarette smoking during pregnancy is associated with downregulation of miR-16, miR-21, and miR-146a in the placenta. Epigenetics.

[112] Tsai K.W., Kao H.W., Chen H.C., Chen S.J., Lin W.C. (2009). Epigenetic control of the expression of a primate-specific microRNA cluster in human cancer cells. Epigenetics.

[113] Muralimanoharan S., Maloyan A., Mele J., Guo C., Myatt L.G., Myatt L. (2012). MIR-210 modulates mitochondrial respiration in placenta with preeclampsia. Placenta.

[114] Ishibashi O., Ohkuchi A., Ali M.M., Kurashina R., Luo S.S., Ishikawa T., Takizawa T., Hirashima C., Takahashi K., Migita M. (2012). Hydroxysteroid (17-beta) dehydrogenase 1 is dysregulated by miR-210 and miR-518c that are aberrantly expressed in preeclamptic placentas: A novel marker for predicting preeclampsia. Hypertension.

[115] Gao W.L., Liu M., Yang Y., Yang H., Liao Q., Bai Y., Li Y.X., Li D., Peng C., Wang Y.L. (2012). The imprinted H19 gene regulates human placental trophoblast cell proliferation via encoding miR-675 that targets Nodal Modulator 1 (NOMO1). RNA Biol.

[116] Maccani M.A., Padbury J.F., Marsit C.J. (2011). miR-16 and miR-21 expression in the placenta is associated with fetal growth. PLoS One.

[117] Zhang Y., Diao Z., Su L., Sun H., Li R., Cui H., Hu Y (2010). MicroRNA-155 contributes to preeclampsia by down-regulating CYR61. Am. J. Obstet. Gynecol.

[118] Wang Y., Fan H., Zhao G., Liu D., Du L., Wang Z., Hu Y., Hou Y. (2012). MiR-16 inhibits the proliferation and angiogenesis-regulating potential of mesenchymal stem cells in severe pre-eclampsia. FEBS J.

[119] Nadeem L., Munir S., Fu G., Dunk C., Baczyk D., Caniggia I., Lye S., Peng C. (2011). Nodal signals through activin receptor-like kinase 7 to inhibit trophoblast migration and invasion: Implication in the pathogenesis of preeclampsia. Am. J. Pathol.

[120] Munir S., Xu G., Wu Y., Yang B., Lala P.K., Peng C. (2004). Nodal and ALK7 inhibit proliferation and induce apoptosis in human trophoblast cells. J. Biol. Chem.

[121] Yu L., Li D., Liao Q.P., Yang H.X., Cao B., Fu G., Ye G., Bai Y., Wang H., Cui N. (2012). High levels of activin a detected in preeclamptic placenta induce trophoblast cell apoptosis by promoting nodal signaling. J. Clin. Endocrinol. Metab.

[122] Xu S., Linher-Melville K., Yang B.B., Wu D., Li J. (2011). Micro-RNA378 (miR-378) regulates ovarian estradiol production by targeting aromatase. Endocrinology.

[123] Lee D.Y., Deng Z., Wang C.H., Yang B.B. (2007). MicroRNA-378 promotes cell survival, tumor growth, and angiogenesis by targeting SuFu and Fus-1 expression. Proc. Natl. Acad. Sci. USA.

[124] Keniry A., Oxley D., Monnier P., Kyba M., Dandolo L., Smits G., Reik W. (2012). The H19 lincRNA is a developmental reservoir of miR-675 that suppresses growth and Igf1r. Nat. Cell Biol.

[125] Forbes K., Westwood M., Baker P.N., Aplin J.D. (2008). Insulin-like growth factor I and II regulate the life cycle of trophoblast in the developing human placenta. Am. J. Physiol. Cell Physiol.

[126] Segura M.F., Hanniford D., Menendez S., Reavie L., Zou X., Alvarez-Diaz S., Zakrzewski J., Blochin E., Rose A., Bogunovic D. (2009). Aberrant miR-182 expression promotes melanoma metastasis by repressing FOXO3 and microphthalmia-associated transcription factor. Proc. Natl. Acad. Sci. USA.

[127] Chelbi S.T., Vaiman D. (2008). Genetic and epigenetic factors contribute to the onset of preeclampsia. Mol. Cell Endocrinol.

[128] Nadeem L., Brkic J., Chen Y.F., Bui T., Munir S., Peng C (2012). Cytoplasmic mislocalization of p27 and cdk2 mediates the anti-migratory and anti-proliferative effects of Nodal in human trophoblast cells. J. Cell Sci.

[129] Caniggia I., Mostachfi H., Winter J., Gassmann M., Lye S.J., Kuliszewski M., Post M. (2000). Hypoxia-inducible factor-1 mediates the biological effects of oxygen on human trophoblast differentiation through TGFbeta(3). J. Clin. Invest.

[130] Oh S.P., Yeo C.Y., Lee Y., Schrewe H., Whitman M., Li E. (2002). Activin type IIA and IIB receptors mediate Gdf11 signaling in axial vertebral patterning. Genes Dev.

[131] Pang R.T., Leung C.O., Ye T.M., Liu W., Chiu P.C., Lam K.K., Lee K.F., Yeung W.S. (2010). MicroRNA-34a suppresses invasion through downregulation of Notch1 and Jagged1 in cervical carcinoma and choriocarcinoma cells. Carcinogenesis.

[132] Montenegro D., Romero R., Kim S.S., Tarca A.L., Draghici S., Kusanovic J.P., Kim J.S., Lee D.C., Erez O., Gotsch F. (2009). Expression patterns of microRNAs in the chorioamniotic membranes: A role for microRNAs in human pregnancy and parturition. J. Pathol.

[133] Yang W.J., Yang D.D., Na S., Sandusky G.E., Zhang Q., Zhao G. (2005). Dicer is required for embryonic angiogenesis during mouse development. J. Biol. Chem.

[134] Red-Horse K., Kapidzic M., Zhou Y., Feng K.T., Singh H., Fisher S.J. (2005). EPHB4 regulates chemokine-evoked trophoblast responses: A mechanism for incorporating the human placenta into the maternal circulation. Development.

[135] Chennakesava C.S., di Santo S., Ziemiecki A., Schneider H., Andres A.C. (2006). Differential expression of the receptor tyrosine kinase EphB4 and its ligand Ephrin-B2 during human placental development. Placenta.

[136] Wang W., Feng L., Zhang H., Hachy S., Satohisa S., Laurent L.C., Parast M., Zheng J., Chen D.B. (2012). Preeclampsia up-regulates angiogenesis-associated microRNA (*i.e*., miR-17, -20a, and -20b) that target ephrin-B2 and EPHB4 in human placenta. J. Clin. Endocrinol. Metab.

[137] Burton G.J., Charnock-Jones D.S., Jauniaux E (2009). Regulation of vascular growth and function in the human placenta. Reproduction.

[138] Muramatsu F., Kidoya H., Naito H., Sakimoto S., Takakura N. (2013). microRNA-125b inhibits tube formation of blood vessels through translational suppression of VE-cadherin. Oncogene.

[139] Alpini G., Glaser S.S., Zhang J.P., Francis H., Han Y., Gong J., Stokes A., Francis T., Hughart N., Hubble L. (2011). Regulation of placenta growth factor by microRNA-125b in hepatocellular cancer. J. Hepatol.

[140] Liu L.Z., Li C., Chen Q., Jing Y., Carpenter R., Jiang Y., Kung H.-F., Lai L., Jiang B.-H. (2011). MiR-21 induced angiogenesis through AKT and ERK activation and HIF-1alpha expression. PLoS One.

[141] Fang L., Deng Z., Shatseva T., Yang J., Peng C., Du W.W., Yee A.J., Ang L.C., He C., Shan S.W. (2011). MicroRNA miR-93 promotes tumor growth and angiogenesis by targeting integrin-beta8. Oncogene.

[142] Zhu J., Motejlek K., Wang D., Zang K., Schmidt A., Reichardt L.F. (2002). β8 integrins are required for vascular morphogenesis in mouse embryos. Development.

[143] Babawale M.O., van Noorden S., Pignatelli M., Stamp G.W., Elder M.G., Sullivan M.H. (1996). Morphological interactions of human first trimester placental villi co-cultured with decidual explants. Hum. Reprod.

[144] Dunk C., Petkovic L., Baczyk D., Rossant J., Winterhager E., Lye S. (2003). A novel *in vitro* model of trophoblast-mediated decidual blood vessel remodeling. Lab. Invest.

[145] Zhao C., Dong J., Jiang T., Shi Z., Yu B., Zhu Y., Chen D., Xu J., Huo R., Dai J. (2011). Early second-trimester serum miRNA profiling predicts gestational diabetes mellitus. PLoS One.

[146] Higashijima A., Miura K., Mishima H., Kinoshita A., Jo O., Abe S., Hasegawa Y., Miura S., Yamasaki K., Yoshida A. (2013). Characterization of placenta-specific microRNAs in fetal growth restriction pregnancy. Prenat. Diagn.

[147] Sibai B., Dekker G., Kupferminc M. (2005). Pre-eclampsia. Lancet.

[148] Faye-Petersen O.M. (2008). The placenta in preterm birth. J. Clin. Pathol.

[149] Cui Y., Wang W., Dong N., Lou J., Srinivasan D.K., Cheng W., Huang X., Liu M., Fang C., Peng J. (2012). Role of corin in trophoblast invasion and uterine spiral artery remodelling in pregnancy. Nature.

[150] Morales Prieto D.M., Markert U.R. (2011). MicroRNAs in pregnancy. J. Reprod. Immunol.

[151] Gilad S., Meiri E., Yogev Y., Benjamin S., Lebanony D., Yerushalmi N., Benjamin H., Kushnir M., Cholakh H., Melamed N. (2008). Serum microRNAs are promising novel biomarkers. PLoS One.

[152] Kotlabova K., Doucha J., Hromadnikova I. (2011). Placental-specific microRNA in maternal circulation—Identification of appropriate pregnancy-associated microRNAs with diagnostic potential. J. Reprod. Immunol.

[153] Chim S.S., Shing T.K., Hung E.C., Leung T.Y., Lau T.K., Chiu R.W., Lo Y.M. (2008). Detection and characterization of placental microRNAs in maternal plasma. Clin. Chem.

[154] Miura K., Miura S., Yamasaki K., Higashijima A., Kinoshita A., Yoshiura K., Masuzaki H. (2010). Identification of pregnancy-associated microRNAs in maternal plasma. Clin. Chem.

[155] Pollheimer J., Knofler M. (2005). Signalling pathways regulating the invasive differentiation of human trophoblasts: A review. Placenta.

